# The Issues of Interoperability and Data Connectedness for Public Health

**DOI:** 10.1089/big.2022.0207

**Published:** 2022-09-06

**Authors:** Laurie T. Martin, Christopher Nelson, Douglas Yeung, Joie D. Acosta, Nabeel Qureshi, Tara Blagg, Anita Chandra

**Affiliations:** ^1^ RAND Corporation, Arlington, Virginia, USA.; ^2^ RAND Corporation, Santa Monica, California, USA.; ^3^ Pardee RAND Graduate School, Santa Monica, California, USA.

**Keywords:** equity, health, public, interoperability, governance

## Abstract

An unprecedented amount of data is being collected across a diversity of sectors, which, if harnessed, could transform public health decision-making. Yet significant challenges stand in the way of such a vision, including the need to establish standards of data sharing and interoperability, the need for innovation in both methodological approaches and workforce models, and the need for data stewardship and governance models to ensure the protection and integrity of the public health data system. As with other articles in this supplement, this article builds from a literature review, environmental scan, and deliberations from the National Commission to Transform Public Health Data Systems. The article summarizes some of the challenges around data sharing and reuse and identifies where the technology and data sectors can contribute to fill current gaps to promote interoperability and data stewardship.

## Introduction

In today's information age, a vast amount of data is collected, and a nontrivial amount of these data hold significant potential for providing a more accurate and timely understanding of the health and well-being of our communities. Yet despite the availability of these data, most remain underutilized or unused given how the data are collected, stored, and structured. The COVID-19 pandemic further exposed the lack of an agile system, with many public health departments so antiquated that relevant forms were filled out by hand and faxed to federal and state officials.^1^

Others did not collect data on race or ethnicity.^2^ And given that data on COVID-19 infections, hospitalizations, and deaths^3^ were not uniformly defined or coded, aggregation and sharing of data within and across communities to provide real-time regional, state, and national impacts was a significant challenge. Addressing these shortfalls, however, goes well beyond the need for additional and sustained resources to collect the best and most timely data. It also requires diverse expertise across sectors to push data equity solutions, develop methodological advances, and adopt core principles of data governance and stewardship that facilitate more widespread data sharing and reuse for the common good.

In this article, we examine the tensions and trade-offs in aspects of data modernization efforts related to interoperability, data stewardship, and governance that could be more widely leveraged in public health. We conclude with a discussion of the skills, expertise, and assets that the data science and technology sectors could bring to questions of data governance and operations to develop a modern equity-oriented public health data system.

## Methods

In 2020, the Robert Wood Johnson Foundation formed the National Commission to Transform Public Health Data Systems to review significant challenges to the current public health data system and “provide recommendations to policymakers, health care organizations and institutions, service providers, and philanthropy” on potential solutions to overcome these challenges.^4^

In support of this effort, RAND conducted an environmental scan to identify key issues, points of consideration, trade-offs and tensions, and current activities related to public health data, data systems, and data modernization efforts. This effort included a targeted scan of published research articles and reports, reviews of websites and working documents describing coordinated activities (e.g., data interoperability), and recent initiatives. Additional searches included the use of “big data” in public health, data privacy, and ethics of public health data collection. Although the team primarily focused on public health data, it also identified seminal articles and reports from other sectors or disciplines whose findings could apply to public health data systems.

RAND simultaneously conducted semistructured interviews with 112 experts and thought leaders on the main topics before the Commission. Individuals represented diverse sectors, including public health and health care, technology and data science, research and policy, journalism, and law. The interviews also included experts in data, data use, equity, community engagement, and research translation who work *outside* the traditional health sector. The project was reviewed and approved by the RAND Human Subjects Protection Committee.

In this article, we highlight relevant findings from this supporting analysis and then implications for the data science and technology sectors, with consideration of recommendations that emerged from the final Commission report.

## Findings

Achieving an equity-centered data system requires more than conceptual buy-in of its importance. Although this is critical in ensuring collective action toward a shared goal, there are numerous practical and logistical challenges that need to be addressed given the current state of public health data systems. Several themes emerged from the literature and stakeholder inputs, as well as Commission deliberations, related to these tensions and include the need to establish standards of data sharing and interoperability; the need for innovation in both methodological approaches and workforce models; and the need for data stewardship and governance models to ensure the protection and integrity of the public health data system.^4,5^

### Models of interoperability and standards of data sharing are not leveraged as widely in public health as in other sectors

The lack of data standards and the sheer number of unique information systems, most of which are not interoperable, pose major technical challenges for efficient and effective use of public health data. To access and integrate data for public health purposes, metadata on available data sets are needed. For example, the Centers for Disease Control and Prevention (CDC) has cataloged their data sets and the metadata and searchable system are available to CDC researchers on the CDC intranet.^6^ Public health more broadly needs a similar set of metadata to be able to efficiently and effectively use the wide array of other data sets in key sectors that influence health, well-being, and equity outcomes.

Given the sheer volume of data and measures currently collected, there may be lessons the public health sector can learn from the technology sector's use of minimum viable product, which promotes agility by allowing users to augment an initial simple set of features to achieve the vision of the product.^7^ As noted in the article on the content of public health data on parsimony, adopting a “less is more” approach for public health data requires some consensus about what bare minimum or core basic set of public health data should be available to all public health departments to ensure they can work toward identified public health priorities. Clear guidance for how to construct the minimum data set would need to be developed to support implementation of such an approach in the future.

One critical consideration for interoperability is how to structure a public health data system that allows for local flexibility, while also ensuring that data collected at a local level can be easily aggregated with data collected elsewhere.^5^ Modularity is a “general systems concept that describes the degree to which a system's components can be separated and recombined and refers to…the degree to which the rules of the system architecture enable or prohibit the mixing or matching of components.”^8^ Modularity can be contrasted with the consolidation or integration of systems, which is another approach to ensuring interoperability, but one that provides less flexibility to meet emerging or unique needs. Although the health care system (and by default health care data), in general, is moving toward consolidation and integration, it is reasonable to ask whether modularity, which has been leveraged successfully in other industries, may be a helpful construct when thinking about the public health data system.^8^

Policies, incentives, and collaborations help catalyze interoperability, but these elements remain fragmented, which can create further inequities in the public health data system.^5^ In recent years, the federal government has leveraged incentive programs to promote interoperability and the collection of a standardized set of data through the Centers for Medicare and Medicaid Services' (CMS) Meaningful Use program^9^ and, more recently, the Merit-based Incentive Payment System.^10^ These incentive programs have tied provider payments to standards of data capture and information exchange.^11^ In 2020, CMS finalized a rule to advance interoperability by promoting the flow of electronic health information (EHI) and providing patients with access to their health information.^11,12^

And in 2020, the Office of the National Coordinator for Health Information Technology released the Cures Act Final Rule “designed to drive interoperability of EHI by supporting the use of … Fast Healthcare Interoperability Resources (FHIR) standards for application programming interfaces (APIs).”^11,13^ Use of Fast Healthcare Interoperability Resources has broad federal support and fosters data sharing between a wide range of potential users, including patients, providers, and other health care entities. However, such incentives could result in inequitable data systems that omit populations who do not see a provider, or who see a provider who does not participate in CMS incentive programs.

Beyond electronic health records (EHRs), several forums and collaboratives have coalesced around issues of interoperability and data sharing across diverse sectors.^14–18^ Collectively, they offer readily available expertise, experience, and lessons learned that could be leveraged to further tackle existing and emerging challenges related to interoperability and standardization. The technology and data sector could also be an important partner in thinking through interoperability considerations because many companies have interoperability baked into their business model.^5^

### Rapidly emerging technology for data collection, analysis, and reuse holds promise, but will require methodological innovation

The integration of data from a wide range of sources, the sheer volume of health-related data being generated including from social media, sensor technology, and new sources of audio and video data, and increased computing power and technological innovation hold great promise for the development of proactive data-driven solutions to improve health, equity, and well-being.^19^ With these changes, however, comes a need for new methodologies to analyze the data efficiently, cost-effectively, and accurately. For example, methodologies for creating robust national estimates from EHR data have yet to be developed.^20^ Furthermore, there is an opportunity to leverage and refine innovations such as artificial intelligence, machine learning, natural language processing, and other methodologies for predictive analytics and the generation of actionable solutions.

To ensure that data governance and protection of privacy keep up with the pace of information technology (IT) innovation, methodological advancements with respect to the de-identification of data that lessen the likelihood of re-identification could also be explored.^21^ Methodological approaches to allow for the disaggregation and analysis of data by geography or population characteristics could also have far-reaching implications for advancing health equity.^5^ Currently, data are either omitted or aggregated with other data if the number of individuals within a “cell” are too small, for the purpose of protecting the privacy of individuals. Oversampling of specific populations or geographies is one strategy for overcoming this challenge, although this approach is resource intensive. Approaches that utilize data from multiple years or introduce noise in the data have also been leveraged to examine populations of interest with smaller numbers, but each has its corresponding limitations.

At the same time, more advanced data collection methods and methodologies must also be critically examined as they could potentially exacerbate inequities in data representation if, for example, existing data or data collection methods designed for one population are applied to another without consideration of whether such approaches are fully appropriate or representative of new spaces, cultures, or populations.^22–25^ Another potential challenge may arise in the development of methodologies, including algorithms, where developers establish benchmarks for acceptability. An accuracy level of 90% may be considered strong, but results in data that is more precise (useful) for some groups and less precise for others.^5^

Privacy protections, particularly of personally identifiable information, are important for ensuring that individuals are not harmed by the inadvertent release of information. At the same time, overly restrictive privacy protections can be problematic for public health or other beneficial reuse cases, because data are either not released or not released in a way that would allow for maximum public benefit.^5^ How data are collected, stored, accessed, and analyzed have also evolved in recent years, and privacy laws vary considerably by state, have not kept pace with rapid advancements in health IT and data management, and do not address “shadow health records,” which are “collections of health data outside the health systems that provide detailed pictures of individual health.”^26^

### Shifts toward greater data sharing and access, both within public health and across diverse sectors, highlight the need for agreed-upon principles of data stewardship and governance

Data stewardship can be thought of both as an institutional commitment to and a collection of methods for data management that address the acquisition, storage, and aggregation of data for scientific and societal benefit, while protecting against privacy and security breaches and misuse.^27^ Data stewardship and governance, however, have not kept pace with rapidly emerging technologies that have an impact on data collection, access, and use.^5^ Issues related to data ownership, access, and trust remain unclear; there is a need for methodological advances to facilitate use of data for public benefit, and there is an opportunity to reassess the necessary competencies of the public health workforce with respect to data stewardship and reuse of data for public good.

The issue of data ownership is perhaps one of the biggest challenges to address in the context of data use for public good.^5^ There is a case to be made that data are owned by the entity that paid for or authorized its collection (e.g., government), such as with state or national surveys. Another position may be that the entity who collected the data (e.g., health care providers, researchers, and private industry) own the health data they collected, given the resources and time spent collecting it, and the competitive advantage such data may provide within their industry.

A third position is that data are owned by the individual providing it, particularly given that in many cases the data contain personal information about that individual. In the context of a public health data system, the issue of ownership becomes particularly important, given that data ownership is likely to vary depending on the type of data, method of data collection, whether consent has been provided, and for what purpose. As data get pooled and shared more readily, the question of ownership becomes even more important because it points to who can grant permission to access the data and for what purpose, and who should profit (financially or otherwise) from the use, sharing, or even selling of data.^5^

If the system is successfully built on the premise of data reuse for public good, knowledge that the data were used for financial gain or competitive advantage could quickly undermine the trust of the public and participating sectors. From an equity perspective, the question of data ownership points to who has the “right” to tell the story stemming from the data, and whether there should be special provisions for where and how that story is told, particularly for special populations.^5^

## Implications

Throughout our analyses and Commission deliberations, it not only became clear that significant practical and logistical challenges will need to be solved, but that data science and technology sector engagement will be critical to helping to solve them. For instance, several of the Commission's recommendations focused on sharing and pooling data and building efficient and interoperable data systems with adequate granularity to generate complete comprehensive and timely data.^4^ Furthermore, the Commission noted that technology companies have an opportunity to support public health data system transformation through sharing of data or financial support.^4^ We highlight four major implications for this sector here.

### Data science and technology companies can inform models and structures of data governance and stewardship

Changes to the types, volume, and greater disaggregation of data to support data-driven solutions to public health challenges will require equitable models of data governance and stewardship. Data science and technology companies are far ahead of public health in establishing best practices and have thought about trust, privacy, confidentiality, and security of data systems. While the 2020 Federal Data Strategy lays out a series of principles, practices, and actions to improve the federal government's approach to stewarding data and using data for public good,^28^ the data science and technology sector is well-poised to help inform these deliberations more broadly within public health, sharing best practices and lessons learned.

### Data science and technology companies can help to build efficient and interoperable data systems

Data science and technology companies continue to be on the cutting edge of information exchange. As such, the data science and technology sector has the expertise to help mitigate and solve challenges within legacy data systems. Continual upgrades to new hardware and software systems are rare among public health departments working within a resource-constrained environment and a workforce whose expertise is focused on health, rather than technology. In addition, to increase accuracy and efficiency, there is a need to develop processes for timely data sharing that require minimal human effort.

### Data science and technology companies could develop agile analytical methods to work with diverse sets of quantitative and qualitative data, including historical data

The integration of data from a wide range of sources coupled with increased computing power and technological innovation hold promise for the development of proactive data-driven solutions to improve health, equity, and well-being.^4,19^ With these changes, however, comes a need for new methodologies to manipulate and analyze data in a way that is not only accurate and efficient, but also balances public health's interest in disaggregation of data while minimizing the likelihood of re-identification.^5,20,21^ Methodological approaches to allow for the disaggregation and analysis of data by geography or population characteristics could also have far-reaching implications for advancing health equity.

### Data science and technology companies could support public health data system transformation through corporate social responsibility or skills-based volunteer approaches

Technology companies' interest in health continues to grow with health-related data being collected from smartphones, wearables, and medical devices. Only a small fraction of these data, however, are consistently used for the public good to identify emerging health needs or to inform local decision-making. Technology companies also have a wealth of talent and are often at the cutting edge of new technologies and approaches to finding signal value within vast amounts of data.

Such analytical skills are often lacking within public health departments, particularly at the local level and within smaller geographic areas.^4,5^ At the same time, there is a larger political and societal question about the role of big technology companies such as Google, Facebook, Microsoft, and Twitter. The public conversations and philosophical questions about how technology companies should behave and what their role in society should be may open a new door for companies to leverage their data, resources, and expertise for public good, and to become powerful allies in crafting a modern equity-oriented data system.^4,5^

## Conclusion

Achieving transformation of the public health data system requires a diverse set of skills and expertise not found within public health alone. The data science and technology sector brings a deep understanding of the practical and logistical challenges of data governance, stewardship, and interoperability, and the diverse talent to develop innovative new methods to help balance the need for greater disaggregation of data with privacy and data security. At the same time, there is a unique opportunity to leverage the sector's expertise and data for the common good and to have a meaningful impact on the health and well-being of their communities.

## Abbreviations Used

CDC: Centers for Disease Control and Prevention
CMS: Centers for Medicare and Medicaid Services’
EHI: electronic health information
EHRs: electronic health records
IT: information technology
